# *CAPN5* gene silencing by short hairpin RNA interference

**DOI:** 10.1186/1756-0500-7-642

**Published:** 2014-09-12

**Authors:** Nnamdi G Nelson, Jessica M Skeie, Hakim Muradov, Hannah A Rowell, Seongjin Seo, Vinit B Mahajan

**Affiliations:** Department of Ophthalmology and Visual Sciences, The University of Iowa Hospitals & Clinics, 200 Hawkins Drive, Iowa City, IA 52242 USA; Omics Laboratory, The University of Iowa Hospitals & Clinics, Iowa City, IA USA

**Keywords:** Autosomal dominant neovascular inflammatory vitreoretinopathy, ADNIV, *CAPN5*, shRNA, Gene therapy

## Abstract

**Background:**

The purpose of this project was to identify short hairpin RNA (shRNA) sequences that can suppress expression of human *CAPN5* in which gain-of-function mutants cause autosomal dominant neovascular inflammatory vitreoretinopathy (ADNIV). We created HEK293T cells that stably express an ADNIV disease allele, *CAPN5*-p.R243L. Transfection protocols were optimized for neuroblastoma SHSY5Y cells. The gene silencing effect of four different shRNA plasmids that target *CAPN5* was tested. RNA and protein expression was determined using quantitative RT-PCR and immunoblot analysis.

**Findings:**

Two of four shRNA plasmids reduced mutant *CAPN5* RNA in a stable cell line. Similar knockdown was observed in SH-SY5Y cells that natively express *CAPN5*. Lactose dehydrogenase assays showed that down-regulation of *CAPN5* was not cytotoxic.

**Conclusions:**

*CAPN5* expression can be suppressed by shRNA-based RNA interference. Further testing in ADNIV models will determine the potential of gene silencing as a strategy to treat, delay, or prevent blindness in ADNIV patients.

## Background

*CAPN5* mutations are the cause of Autosomal Dominant Neovascular Inflammatory Vitreoretinopathy (ADNIV, OMIM #602537), an inherited autoinflammatory uveitis and vitreoretinal degeneration without systemic features [1]. ADNIV patients are normally sighted until their second decade when a chronic uveitis begins and cataracts develop. Over the next five decades, photoreceptor degeneration, retinal neovascularization and intraocular fibrosis occur sequentially and end in phthisis and blindness [1, 2].

*CAPN5* encodes calpain-5, a calcium-activated cysteine protease [3]. A single allele containing a nonsynonymous mutation (*CAPN5*-p.R243L) is sufficient to cause ADNIV [2, 4, 5]. The mutation is located within a gatekeeper domain that controls access to the active site [1]. The *CAPN5*-p.R243L mutant allele is predicted to disrupt the gatekeeper domain and increase the calpain-5 proteolytic activity. This gain of function model is consistent with the mode of inheritance, high penetrance, disease severity, and recent allele testing in a mouse model [6]. Therapies that target secondary downstream effects are insufficient to reverse disease [5], so therapies aimed at the primary genetic defect are necessary.

Gene therapy for retinal diseases is feasible. Gene replacement therapy was successfully applied in humans for the autosomal recessive retinal degeneration associated with *RPE65* Leber’s Congenital Amaurosis [7, 8]. Gene silencing therapy, however, is required to treat autosomal dominant gain of function diseases such as ADNIV. Suppression of gene expression may be sufficient to limit the *CAPN5* disease allele physiological activity and delay disease onset and progression. RNA-based gene silencing is one approach that has been applied to mouse and cell models of similar dominantly inherited retinal degenerations [9, 10]. RNA-based gene silencing has also been applied in cases of cytomegalovirus retinitis in patients with AIDS and in age-related macular degeneration [11], supporting this general strategy for human retinal therapy.

RNA-based gene silencing methods generate gene-specific complementary RNA sequences that form double stranded RNA within cells. Short hairpin RNAs (shRNAs), for example, are composed of a sense and antisense sequence connected by a loop of unpaired nucleotides [12]. shRNA binds to a target sequence, an RNase engaging DICER cleaves the shRNA into siRNA, and the Argonaute (AGO) protein cleaves the siRNA target sequence. A mature RNA-induced silencing complex (RISC) that contains a guide strand and an AGO protein directs the complex to associate with the target mRNA for degradation or post-transcriptional gene silencing [12, 13]. The purpose of this study was to develop a disease-allele cell line, identify shRNA sequences that reduce calpain-5 expression, and deliver these to cells that natively express *CAPN5.*

## Findings

### CAPN5 stable cell line

**Figure 1:**
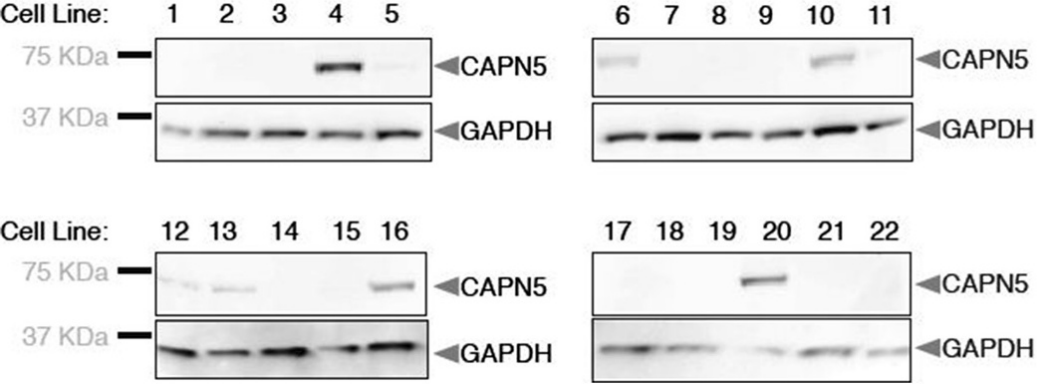
**Calpain-5 is stably expressed in HEK293T cells.** HEK293T cells were co-transfected with *CAPN5*-p.R243L and puromycin plasmids and twenty-two clones were analyzed. Calpain-5 protein expression was detected with an anti-myc antibody in protein extracts (upper panels). GAPDH levels are indicated (lower panels). A HEK293T cell line that showed moderate expression (lane 16) was propagated for use in the silencing experiments.

To study the *CAPN5*-p.R243L disease allele in cultured cells, we generated a stably expressing cell line using HEK293T cells, which do not normally express calpain-5. Immunoblot analyses of twenty-two cell colonies revealed that seven of them successfully expressed the mutant *CAPN5* (Figure 1). A moderate expression colony (lane 16) was selected for further experimentation. After several passages, immunoblot verified that this line continuously expressed *CAPN5-*p*.*R243L.

### Transfection optimization

**Figure 2:**
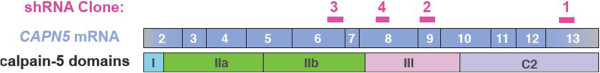
**shRNA sequence targeting map.** A. Schematic of the human *CAPN5* gene and the alignment of four candidate anti-human *CAPN5* shRNAs. The *CAPN5* is composed of twelve exons that code for the four domains of calpain-5.

**Figure 3:**
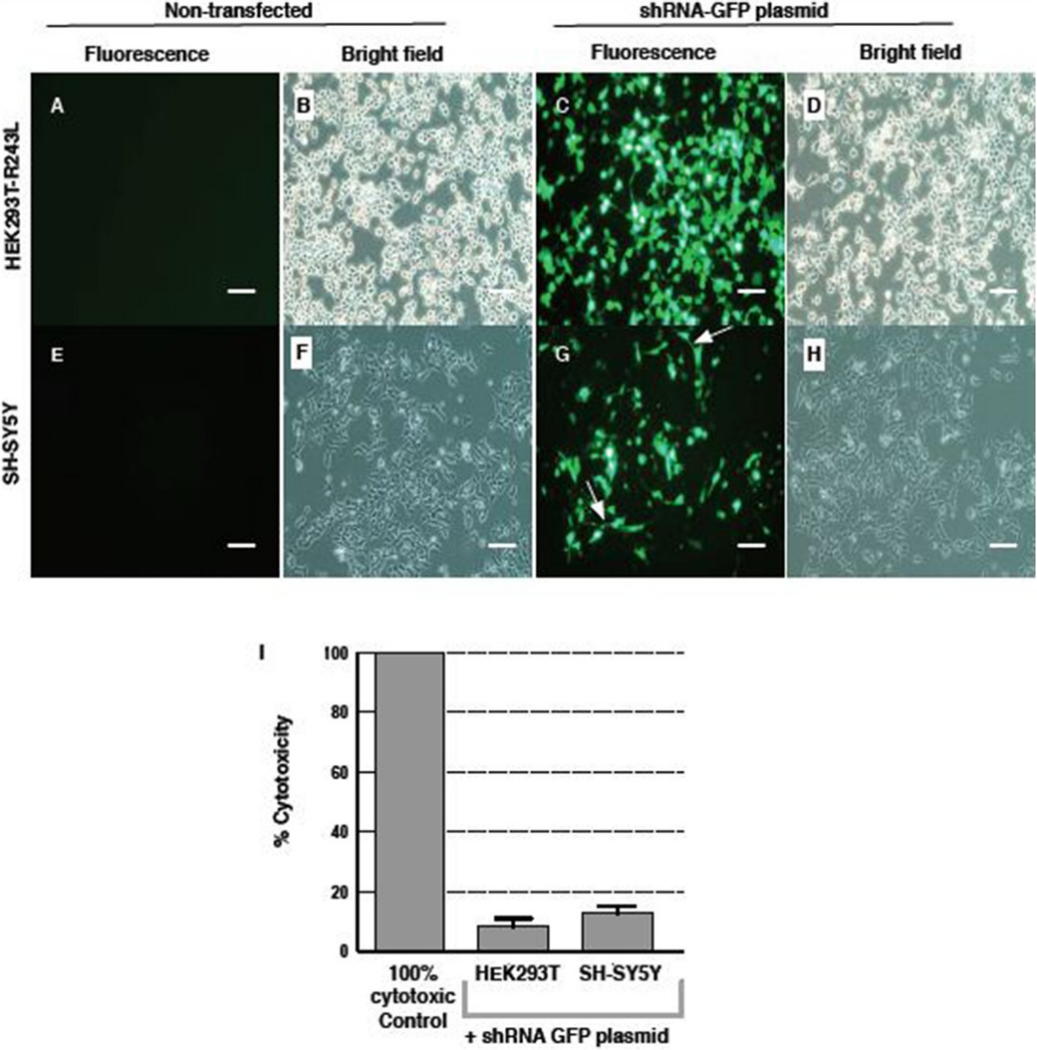
**Cells express GFP after transfection with plasmids.** We tested the transfection of shRNA-GFP plasmid in two cell types, HEK293T-*CAPN5*-p.R243L **(A-** **D)** and SH-SY5Y **(E-** **H)**. **C**, **G**. HEK293T-*CAPN5*-p.R243L and SH-SY5Y neuroblastoma cells successfully transfected with negative control shRNA plasmid expressed GFP marker. In **G**. arrows show short neuritis extend from cell. **A**, **E**. Cells not transfected with plasmid show no background labeling under fluorescent light. **B**, **D**. Under bright field, HEK293T-*CAPN5*-p.R243L cells proliferate as an adherent layer. **F**, **H**. SH-SY5Y cells proliferate as an adherent clump. Cells were visualized under 20X magnification. Scale bars: 100 μm. **I**. As revealed by LDH assay, the knockdown of *CAPN5* was not cytotoxic.

Four shRNA sequences targeting different human *CAPN5* exons were selected for testing (Figure 2). HEK293-R243L cells were transfected with the shRNA plasmids using a lipid reagent and showed over 90% transfection efficiency (Figure 3) and good cell viability (data not shown). Cells expressing GFP were observed after 48 hours incubation (Figure 3). In contrast to HEK293T cells, the human neuroblastoma cell line SH-SY5Y expresses native *CAPN5* and has been used to study CAPN5 protein expression [14]. The transfection efficiencies of SH-SY5Y cells using lipid-based reagents were less than 10% (data not shown). Using electroporation, we optimized transfection efficiencies in SH-SY5Y cells. The optimal electroporation parameter for transfecting SH-SY5Y cells was 1200 pulse voltage, 20 pulse width and 2 pulses. This gave efficiencies up to 80% in SH-SY5Y (Figure 3). GFP expressing SH-SY5Y cells were observed after 72 hours incubation. Cytotoxicity of the shRNA plasmids treatment was measured using an LDH assay. In all the three cell lines, cytotoxicity of the shRNA clones was minimal (Figure 3I).

### Gene silencing

**Figure 4:**
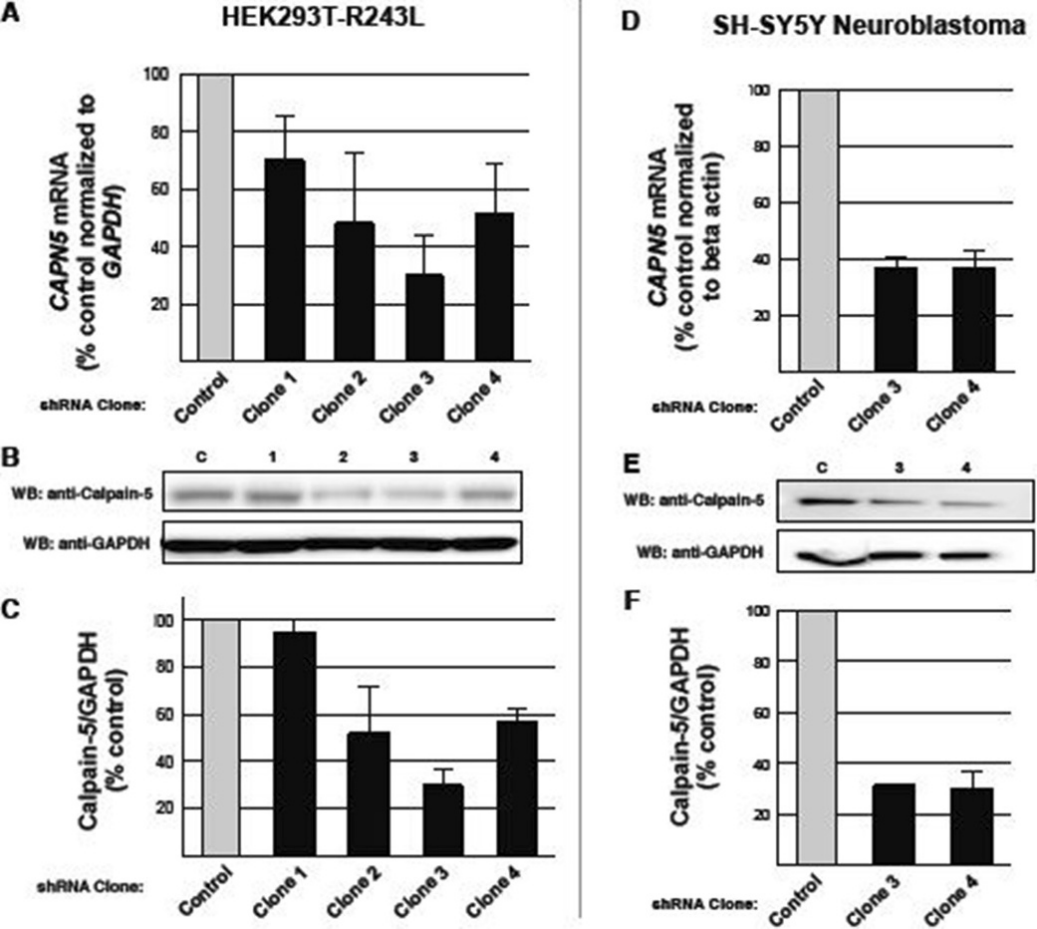
**shRNA suppresses** ***CAPN5*** **in cell lines.** Quantitative PCR and immunoblot results for HEK293T-*CAPN5*-p.R243L **(A-** **C)** and SH-SY5Y **(D-** **F)** show knockdown effect of shRNA transfection. **A**, **D**. *CAPN5* mRNA levels relative to *GAPDH* (HEK293T-*CAPN5*-p.R243L) or β-actin (SH-SY5Y) mRNA after transfection with each shRNA vectors as shown by percent knockdown of the respective vectors compared to the negative control vector. Quantitative PCR data are from average of three independent experiments. **B**, **E**. Immunoblots of transfected cell lysates show calpain-5, with GAPDH immunostaining as the loading control. **C**, **F**. Immunoblot band densities depict the average of three independent experiments. Bar graphs were represented as a mean ± SEM.

To determine which shRNA sequences would diminish *CAPN5*-p.R243L expression, we tested 4 different human sequences and a nontargeting control sequence in HEK293T-R243L cells. The nontargeting control sequence did not show significant suppression of the *CAPN5*-p.R243L mRNA. Plasmid clones 1 and 2, which targeted exons 13 and 9 of the mRNA respectively (Figure 4A), did not significantly suppress *CAPN5* mRNA levels. On the other hand, shRNA clones 3 and 4, which targeted exons 6 and 8 of *CAPN5* mRNA respectively, were able to suppress *CAPN5*-p*.*R243L by over 70% and 50%, respectively (Figure 4A).

Next, we performed immunoblots to measure calpain-5 protein levels. Clones 3 and 4 significantly reduced calpain-*5* expression levels in the HEK293T-R243L cell line (Figures 4B and C). Knockdown was most pronounced with clone 3 plasmid, showing over 70% decrease in both RNA and protein levels, compared to the effect of the negative control.

To determine if these shRNA clones would suppress native *CAPN5*, clones 3 and 4 were used to transfect the human neuroblastoma cell line SH-SY5Y. Again, the nontargeting shRNA did not significantly reduce *CAPN5* mRNA levels, but clones 3 and 4 each reduced expression by approximately 65% (Figure 4D). Immunoblot densities showed that both clones reduced calpain-5 by approximately 70% (Figures 4E and F).

## Discussion

In this study, we identified two shRNA sequences that effectively down-regulate both mutant and native *CAPN5* at the RNA and protein level. The disease allele of *CAPN5* in ADNIV represents a gain-of-function where there is enzymatic over-activity [1]. These data establish shRNA-mediated RNA interference (RNAi) as a potential therapeutic strategy to rescue mutant *CAPN5* disease phenotypes.

RNA interference using shRNA has been successful in animal models of dominantly inherited ocular diseases such as cone-rod dystrophy (retinitis pigmentosa) and age-related macular degeneration [15, 16]. RNA-based gene silencing was also applied to the calpain-5 homolog *tra-3* in the *C. elegans*. RNA inhibition of *tra-3* rescued neurons from necrotic cell death [17]. Since necrotic cell death is associated with inflammation, this may parallel the inflammatory neuronal degeneration in ADNIV and further supports a similar gene silencing strategy to rescue the ADNIV phenotype. ADNIV symptoms do not begin for nearly two decades [1], so there is ample opportunity to intervene before the disease becomes severe. The slow progression of disease also suggests that even partial inhibition of calpain-5 could delay the disease enough to have an impact on vision, especially in combination with other therapies [5]. Our recent development of an ADNIV mouse model provides a preclinical model to test *CAPN5* RNA inhibition *in vivo* [6].

The eye is an optimal organ for gene therapy because it is compartmentalized, which enhances precise gene delivery and reduces systemic dissemination that might trigger immune responses [18]. The compartmentalization of the eye also ensures that resident cells can be transduced effectively with low doses of a therapeutic agent. The numerous non-invasive imaging methods allows easy monitoring of treatment and unwanted side effects of gene therapy [18]. RNAi-based gene therapy may be limited by the difficulty in ensuring efficient delivery and limiting off-target effects. Some of these setbacks have been addressed by the use of recombinant adeno-associated viruses (rAAVs) as an efficient vehicle to deliver shRNA to specific cells or tissues and the use of tissue-specific promoters ensure that only target tissues with specific promoters are able to stably express the therapeutic gene products at effective levels long term [19, 20]. Due to these attributes, experiments and trials employing RNAi-based gene therapy are currently underway for treatment of hereditary ophthalmic pathologies such as retinitis pigmentosa and age-related macula degeneration [11, 21, 22]. Clinical trials for therapy are in the planning stages for primary open angle glaucoma [23]. If gene silencing is effective in an ADNIV animal or cell models, a clinical trial with ADNIV patients is feasible.

## Conclusion

Short Hairpin RNA interference is an effective method for reducing *CAPN5* gene expression and may address other gain-of-function disease alleles that cause human blindness.

## Methods

### Cell culture, reagents and antibodies

Human embryonic kidney 293 T (HEK293T) and differentiated human neuroblastoma SH-SY5Y cells were purchased from the American Type Culture Collection (ATCC; Manassas, VA). Cells were maintained in 90 mm non-pyrogenic tissue culture dishes with 15 mL growth medium at 37°C in a humidified atmosphere with 95% air and 5% CO_2_. Heat-inactivated fetal bovine serum (FBS), penicillin-streptomycin antibiotic, Dulbecco’s Modified Eagle Medium (DMEM) containing 4500 mg/L D-glucose and L-glutamine, without sodium pyruvate, OptiMEM reduced serum medium and RPMI-1640 medium, both containing L-Glutamine, were all purchased from Gibco (Grand Island, NY). Mouse monoclonal anti-GAPDH antibody (6C5), goat anti rabbit secondary antibody and goat anti mouse secondary antibody were purchased from Santa Cruz Biotechnology, Inc. (Santa Cruz, CA); rabbit polyclonal anti-calpain-5 antibody was purchased from GeneTex (Irvine, CA); rabbit anti-myc tag antibody was purchased from Abcam (Cambridge, MA).

HEK293T adherent cells were grown in DMEM medium supplemented with 10% FBS, penicillin (100 U/mL) and streptomycin (100 μg/mL) (complete DMEM medium). The HEK293T cells were transformed into cells that stably express a disease mutant of calpain-5 (p.R243L). SH-SY5Y adherent cells were cultured in a 1:1 mix of RPMI 1640 and F-12 media containing 10% FBS, penicillin (100 U/mL) and streptomycin (100 μg/mL).

### Short hairpin RNA plasmids

Four unique short hairpin RNA (shRNA) sequences that target human *CAPN5* and one nontargeting sequence (negative control) were purchased from Qiagen (Catalog# KM40517; Frederick, MD). Each plasmid vector expressed a shRNA under the control of a U1 promoter and contained a green fluorescent protein (GFP) reporter gene. The shRNA nucleotide sequences were: clone one: 5’ - GTACAATGTGAAAGGCATCTT - 3’; clone two: 5’ - CAACCACAAGGACACCTTCTT - 3’; clone three: 5’ - GACTTCACGGGTGGTGTTTCT - 3’; clone four: 5’ - GTCAGAGAAGTTGGTGTTTCT - 3’; negative control: 5’ - GGAATCTCA TTCGATGCATAC - 3’.

### Stable cell line creation

HEK293T cells were co-transfected with a pUS2 vector that encodes the puromycin-resistant gene and myc-tagged *CAPN5* wild type (WT) or p.R243L (disease-allele) in pCMV6 entry vector (Origene Technologies Inc.; Rockville, MD). The pUS2 vector was modified from pCS2 entry vector by replacing the CMV promoter with UbC promoter. In wells of separate 12-well plates, HEK293T cells at 70% confluence in 600 μL complete culture medium were co-transfected with 900 ng of *CAPN5* and 100 ng of puromycin plasmids using the lipid reagent Turbofectin (OriGene Technologies, Inc.; Rockville, MD) per manufacturers’ recommendation. After 24-hours incubation at 37°C, the cells were trypsinized and resuspended in 1 mL selection medium (comprising of DMEM with 10% FBS, 1% L-glutamine, penicillin (100 U/mL), streptomycin (100 μg/mL) and 1.5 ug/mL puromycin). Briefly, 500 μL of the cell suspension was added into 1.5 mL of fresh selection media per well in new 12-well plate and incubated for 24 hours. The transfected cells were trypsinized, serially diluted and re-plated in separate 10 cm dishes with 10 mL selection media. The cell cultures were maintained for 12–14 days until the colonies attained diameters between 2–4 mm. Finally, sterile forceps and cloning rings dipped in silicone grease were used to select twenty-four distinct colonies by trypsinization, followed by incubation at 37°C for 15 minutes, and then resuspended in 300 μL of selection medium. The cells of each colony were cultured in a new 24-well plate containing 700 μL selection medium per well. Once the cells in each well attained 50-70% confluence, they were seeded and cultured in a new 24-well plate for screening and also in four 6-well plates for maintenance. The cells were screened for calpain-5 using immunoblot. Cells were lysed on ice for 10 minutes with HEPES buffer [50 mM HEPES (pH 7.0), 150 mM NaCl, 2 mM EGTA, 2 mM MgCl_2_, 1% Triton X-100, protease inhibitor cocktail (Roche; Indianapolis, IN)]. Twenty-two cell lysates were collected after centrifugation at 20,000xg for 10 minutes. Equal amounts of protein were blotted and probed with HRP-conjugated rabbit polyclonal antibody to Myc tag (1:5000), mouse monoclonal anti GAPDH (1:400), and secondary goat anti rabbit IgG (1:5000).

### Gene suppression by shRNA plasmid transfection

HEK293T-*CAPN5*-p.R243L cells were transfected using Attractene Transfection Reagent (Qiagen). In each well of 6-well plates, 5.0 × 10^5^ HEK293T-*CAPN5*-p.R243L cells were grown in 2 mL complete DMEM medium to 50 - 70% confluence. Growth medium was replaced with 2 mL of freshly prepared Opti-MEM medium containing 5% FBS, penicillin (100U/mL) and streptomycin (100 μg/mL) (complete Opti-MEM medium). Cells were then transfected with 3.0 μg shRNA plasmid using 4.5 μL Attractene Reagent. At 6 hours post-transfection, growth medium was replaced with fresh complete Opti-MEM medium. Cells were incubated for 48 hours.

The optimization protocols (varying pulse voltage, pulse width and pulse, as recommended by manufacturer) for transfecting adherent and suspension cells with Neon™electroporation system (Invitrogen; Carlsbad, CA) were performed with SH-SY5Y cells. Briefly, 1.0 × 10^6^ SH-SY5Y cells in 100 μL resuspension buffer were electroporated with 12.0 μg shRNA plasmids into 150 × 20 mm tissue culture dishes containing 15 mL Opti-MEM medium containing 5% FBS. After optimization, transfection of SY-SY5Y cells were performed in triplicate for each shRNA plasmid and incubated for 72 hours.

### Lactose dehydrogenase (LDH) assay

Toxicity of *CAPN5* gene suppression on transfected cells was measured by LDH release assay (Cytotoxicity Detection Kit, Roche Applied Science, Mannheim, Germany). LDH activity in the culture medium indicated LDH release from cells. Culture medium was collected from transfected cells after 48 hours incubation. For low cytotoxicity control, cells were cultured without plasmid transfection. For high cytotoxicity control, cells were treated with 1% Triton X-100 in assay medium. Culture media from all conditions was centrifuged at 250xg to remove cell contaminants. The background controls were prepared by mixing transfection medium with Attractene or electroporation buffer. An aliquot (100 μL) of the supernatants and the background control medium were collected into a 96-well plate and incubated with 100 μL of freshly prepared LDH assay reaction mixture. Incubation was performed in the dark for 30 minutes at room temperature. Absorbance was read at 490 nm using an Infinite M200 PRO microplate reader (Tecan Systems Inc.; San Jose, CA). Cytotoxicity was calculated using the equation: Cytotoxicity (%) = [(test sample – low control)/(high control ‒ low control)] × 100.

### Immunoblot

Calpain-5 levels were measured by immunoblot densitometry. Cells were rinsed with 1X PBS and homogenized in RIPA extraction buffer (1% sodium deoxycholate, 0.1% SDS, 1% Triton X-100, 10 mM Tris–HCl, pH 8.0, 0.15 M NaCl, 0.047 M NaF and 5.0 mM EDTA) containing protease inhibitor cocktail from Roche, Indianapolis, IN. Homogenates were sonicated on ice for 10 seconds, two times and then centrifuged at 1000xg for 10 minutes at 4°C. Total cellular protein was measured using the modified Lowry method (*DC* Protein Assay; Bio-Rad), and equal amounts of protein were used. Samples were denatured at 85°C for 5 minutes in LDS-sample buffer with reducing agent (Invitrogen; Carlsbad, CA), and electrophoretically separated using 4-12% NuPAGE Bis-Tris gels. HEK293T-R243L proteins were transferred onto PVDF membranes while SH- SY5Y proteins were transferred onto nitrocellulose membrane. Membranes were blocked at 4°C with 5.0% non-fat dry milk in 1x TBS 0.1% tween-20. The proteins were immunostained with rabbit polyclonal antibody to calpain-5 (GeneTex; 1:1000) and mouse monoclonal antibody to GAPDH (Santa Cruz; 1:400). Goat anti-rabbit-HRP and goat anti-mouse-HRP antibodies (both were used at 1:10,000 dilution ratios) were used as the secondary antibody. The immunoreactive bands were detected with Supersignal West Dura Extended Duration Substrate (Thermo Scientific; Rockford, IL). Visualization and quantification of band intensities were performed with MYECL imager and software (Thermo Scientific). We quantified Calpain-5 expression in three independent experiments by normalizing the calpain-5 to GAPDH and calculating the mean expression in experimental groups (shRNA clones) relative to the control group (negative control shRNA).

### Quantitative real time PCR (qPCR)

*CAPN5* mRNA levels were measured by qPCR. RNA was isolated from the cells using Qiagen RNeasy mini kit. cDNA was prepared from 1 μg of total RNA using High Capacity cDNA Reverse Transcription kit with RNAse Inhibitor (Applied Biosystems Inc., Foster City, CA). Total RNA and cDNA concentrations were measured at 260 and 280 nm using Nanodrop 2000c spectrophotometer (Thermo Scientific). Quantitative PCR was performed with 100 ng of first-strand cDNA in 25 μL reaction mix using Power SYBR Green PCR Master Mix (Applied Biosystems). The primers used were: *CAPN5* forward, 5’-TGATCAACACATCCCACCTG-3’; reverse, 5’-ACGCTGCGTGAGTTGATGTA-3’; *GAPDH* forward, 5’-CGAGATCCCTCCAAAATCAA-3’; reverse, 5’-GTCTTCTGGGTGGCAGTGAT-3’; Beta-actin forward, 5’-ACCCTGAAGTACCCCATTG-3’; reverse, 5’- TACGACCAGAGGCATACAG-3’. Data were normalized to *GAPDH* mRNA for HEK293T-R243L cells and Beta actin mRNA levels for SH-SY5Y cells. The qPCR cycles were: 95°C for 10 min; (95°C for15 s; 60°C for 1 min) × 40 cycles. Comparative threshold cycle (*C*_*t*_) method was used to measure relative gene expression in response to shRNA plasmid treatments. All data analyses were conducted using Prism 6.0 software (GraphPad; La Jolla, CA).
